# Role of GSTM1 and GSTT1 Gene Polymorphisms on Lung Cancer Susceptibility and Effect on Platinum-Based Chemotherapy-Induced Toxicity in Bangladeshi Lung Cancer Patients

**DOI:** 10.1155/jcep/9194714

**Published:** 2024-11-20

**Authors:** Tahsin Nairuz, Yearul Kabir

**Affiliations:** ^1^Department of Biochemistry and Molecular Biology, Noakhali Science and Technology University, Noakhali, Bangladesh; ^2^Department of Biomedical Engineering, Keimyung University, Daegu 42601, Republic of Korea; ^3^Department of Biochemistry and Molecular Biology, University of Dhaka, Dhaka, Bangladesh

**Keywords:** GSTM1, GSTT1, lung cancer, platinum-based chemotherapy, polymorphisms

## Abstract

**Background:** Glutathione S-transferases (GSTs) play a significant role in carcinogen detoxification, and hence, polymorphisms of this gene may lead to lung cancer susceptibility. Accordingly, this study is aimed at investigating GSTM1 and GSTT1 polymorphisms' association with lung cancer risk and their effects on the toxicities of platinum-based chemotherapy used to treat Bangladeshi lung cancer patients.

**Methods:** The study subjects comprised 180 lung cancer patients and 200 healthy volunteers. Genetic polymorphisms of GSTM1 and GSTT1 were analyzed using a multiplex PCR–based method. Chemotherapy toxicity was evaluated using the Common Terminology Criteria for Adverse Events (CTCAE v5.0).

**Results:** GSTM1 null genotype was found to be significantly associated with a 1.6-fold increased risk of lung cancer (OR = 1.60, 95%CI = 1.01–2.52, *p* = 0.0491), whereas no significant association was observed with GSTT1 null genotype and combined GSTM1 and GSTT1 null genotype. Moreover, no significant relationship was observed between GSTM1 and GSTT1 polymorphisms and the increased risk of platinum-based chemotherapy-induced toxicities in lung cancer patients.

**Conclusions:** These findings indicated that the GSTM1 null but not GSTT1 null genotype was significantly associated with lung cancer susceptibility. These polymorphisms were not related to platinum-based chemotherapy-induced toxicities in Bangladeshi lung cancer patients.

## 1. Introduction

Worldwide, lung cancer is the prime cause of cancer-related death among men and women and one of the most frequent malignancies with complex, multifactorial etiology. Although exposure to cigarette smoking is believed to be a significant etiological factor for lung carcinogenesis (accounts for about 90% of the risk of lung cancer in men and 70%–80% in women) [1], not all smokers but many nonsmokers develop lung cancer indicating other aspects such as environmental factors (carcinogen exposure) and most notably genetic factors might play an immense role in individual differences to lung cancer susceptibility.

Glutathione S-transferases (GSTs) consist of a Phase II transformation enzyme superfamily participating in the detoxification of potentially carcinogenic and toxic compounds by conjugating with reduced glutathione, thereby eliminating these compounds and hindering their association with vital nucleic acids and cellular proteins [2]. Among the GSTs, GSTM1 metabolizes and detoxifies preferentially prominent hydrophobic carcinogens (polycyclic aromatic hydrocarbon–derived epoxides) [3]. On the other hand, GSTT1 is engaged in the metabolism and biotransformation of many minor toxin compounds (monohalomethane and ethylene oxide) [4].

Genetic polymorphisms related to decreased activity of GSTs have been of great interest during recent years due to interindividual, demographical, ethnic, and geographical differences in environmentally induced diseases, including cancer susceptibility worldwide. GSTM1 and GSTT1 genes manifest an inherited homozygous deletion polymorphism (null genotype), leading to the lack of enzymatic activity and increased risk for malignancies [5]. In lung tissues, individuals carrying GSTM1 null genotype are claimed to possess increased polycyclic aromatic hydrocarbon dGMP adducts that can trigger genetic mutations [6]. On the other hand, individuals who are GSTT1 null allele carriers may have a decreased capability to excrete carcinogenic particles metabolically and, consequently, be at higher risk of cancer [7, 8].

Drug therapy for lung cancer mostly includes platinum-related chemotherapy regimens that are widely recognized as the first-line treatment for most patients where radiotherapy cannot be used. Besides their influential role in cancer chemotherapy, platinum-related toxicity is prevalent in clinical situations [9]. Evidence supports that genetic polymorphisms promote variations in platinum-induced toxicities [10]. Therefore, exploring the relationship between genetic polymorphisms and platinum-induced toxicities is valuable to individualized chemotherapy. GSTs participate in the detoxification of platinum-based components [11]. Reduced and loss of the activity of polymorphic GSTs may reduce the detoxification of platinum compounds. Previous studies have found that GSTs are linked to resistance to cisplatin-based chemotherapy [12, 13].

In Bangladesh, a relatively high incidence of lung cancer is observed, where it accounts for about 12,174 or 1.70% of total deaths, according to the World Health Organization data, 2020 [14]. It ranks as the most frequent cancer in Bangladeshi males, with an incidence rate of 14.2% [15]. However, only a few studies have been conducted in the Bangladeshi population demonstrating the association of GST (GSTM1 and GSTT1) polymorphisms with the risk of lung cancer having a small sample size. Still, none of these studies revealed the effect of these polymorphisms on platinum-based chemotherapy toxicity. Hence, the present study was designated to investigate the possible correlations of GSTM1 and GSTT1 polymorphisms with lung cancer susceptibility. Moreover, this is the first study to evaluate the impact of these gene polymorphisms on the toxicity of platinum-based chemotherapy in Bangladeshi patients with lung cancer.

## 2. Materials and Methods

### 2.1. Study Subjects and Data Collection

The study was hospital based; the case–control study consisted of 180 lung cancer patients (cases) and 200 healthy volunteers (controls), where patients were histologically proven with lung cancer following the International Association of Lung Cancer [16] and received platinum-based chemotherapy. Briefly, from January 2016 to May 2017, a standard protocol was used to recruit lung cancer patients from three renowned cancer treatment–related hospitals in Bangladesh (Ahsania Mission Cancer and General Hospital, Dhaka Medical College Hospital, and Bangabandhu Sheikh Mujib Medical University). None of the lung cancer cases had records of other serious diseases; however, if identified, they were eliminated from the study. Controls were chosen following physical examination ensuring the absence of any acute or chronic diseases and recruited from the Dhaka University Medical Centre, Department of Biochemistry and Molecular Biology, and Kobi Sufia Kamal Hall, University of Dhaka, Bangladesh Institute of Research and Rehabilitation in Diabetes, Endocrine and Metabolic Disorder (BIRDEM), Popular Diagnostics Center, Dhanmondi, Dhaka. Before enrolment, informed consent was received from all participants, and a structured questionnaire was completed by them containing information on age, gender, smoking history, pathological tumor stage, toxicities of chemotherapy, and family history of chronic diseases. According to the interview, current and ex-smokers were regarded as ever smokers, whereas never smokers were those who had never smoked during their lifetime. The study was conducted following the provisions of the recent Helsinki Declaration [17], and the Ethical Review Committees of the Department of Biochemistry and Molecular Biology, University of Dhaka, approved the protocol.

### 2.2. Toxicity Evaluation Criteria

Toxicities of platinum-based chemotherapy, such as neutropenia, anemia, thrombocytopenia, leukopenia, and gastrointestinal toxicity in lung cancer patients, were evaluated using Version 5.0 Common Terminology Criteria for Adverse Events (CTCAE v5.0) of the National Cancer Institute [18].

### 2.3. Genotyping

**Figure 1:**
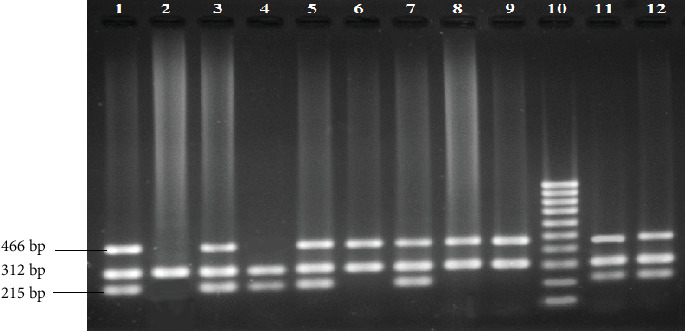
Multiplex PCR analysis of GSTM1 and GSTT1 gene polymorphism. GSTM1 (215 bp), GSTT1 (466 bp), and exon-7 CYP1A1 (312 bp) genes.

About 3.0 mL of venous blood samples was obtained from all participants, maintaining all aseptic precautions in sterile tubes (EDTA–Na_2_ containing) for genotyping study and until DNA extraction, stored at −20°C. The genomic DNA was extracted from peripheral leukocytes following the standardized protocol used in our laboratory [19, 20]. The allele-specific multiplex PCR–based method was performed to identify GSTM1 and GSTT1 genotypes, where a part of the exon-7 CYP1A1 gene was amplified as an internal control, ensuring the absence of either GSTM1 or GSTT1 allele as null genotype instead of the failure of PCR. A negative control was also employed in each PCR reaction to confirm the absence of any contaminants. The primer sequences and PCR conditions for amplifying GSTM1, GSTT1, and exon-7 CYP1A1 fragments were derived from the previously published paper [21]. The coamplified products were resolved by electrophoresis on 2% agarose gel, and the optimal size of the product was determined by comparing it to the DNA ladder. Following staining with ethidium bromide, the amplified DNA was visualized under UV light, and a gel image was captured and documented (Figure 1). The presence of a 215-bp band identified individual homozygote or heterozygote for GSTM1. In contrast, in the presence of the internal control band, its absence indicated GSTM1 null genotype. On the other hand, a 466-bp band identified individual homozygote or heterozygote for GSTT1, whereas its absence indicated GSTT1 null genotype in the presence of the internal control band.

### 2.4. Statistical Analysis

All statistical analyses relevant to this study were performed through the SPSS software (Version 17.0) and GraphPad Prism, Version 8. A chi-square (*χ*^2^) test was done to compare different demographic and clinical characteristics between patients and controls. Fisher's exact test compared the distribution of genotypic frequency. Toxicity variations according to toxicity grades between the various genotypes of GSTM1 and GSTT1 were also evaluated using Fisher's exact tests. The relative associations were estimated by calculating the odds ratio (OR) with 95% confidence intervals (CIs) and level of significance (*p*). *p* < 0.05 was regarded as a level of significance.

## 3. Results

### 3.1. Characteristics of the Study Subjects

The distributions of demographic and clinical characteristics among cases and controls are represented in Table 1. Lung cancer patients were slightly older than controls, with a mean age of 55.83 in cases versus 54.70 in controls, although statistically, no significant differences were found regarding age. On the other hand, in cases, males were overrepresented than females (60.56% vs. 39.44%), indicating that lung cancer was significantly higher in males (*p* < 0.001). Moreover, the frequency of smokers was significantly higher in patients with lung cancer than in controls (71.11% vs. 37.5%, *p* < 0.001). Additionally, in both cases and controls, individuals with no family history of cancer represented significantly higher frequency (*p* < 0.001) than subjects with a previous family history of cancer. All lung cancer patients received platinum-based chemotherapeutic agents combined with some nonplatinum agents, where carboplatin + paclitaxel represented the highest frequency at 31.67% (57 patients) and carboplatin + doxorubicin showed the lowest frequency at 3.33% (6 patients).

### 3.2. Frequency Distribution of GSTM1 and GSTT1 Genotype and Risk of Lung Cancer

GSTM1 and GSTT1 genotype frequencies and estimated risk of lung cancer are shown in Table 2. Among 180 lung cancer patients, 31.67% carried GSTM1 null allele, and 68.33% were GSTM1 positive. On the other hand, 22.50% had GSTM1 null allele in control subjects, and 77.50% were GSTM1 positive. There was a significant difference in GSTM1 null genotype frequency distribution between lung cancer patients and controls. The GSTM1 null genotype was found to be significantly associated with a 1.6-fold increased risk of lung cancer (OR = 1.60, 95%CI = 1.01–2.52, *p* = 0.049).

On the other hand, 20.56% of the patient group carried the GSTT1 null genotype, much less than that of control subjects (26.50%). No significant difference was observed in the frequency distribution of the two groups carrying the null genotype, and the risk of lung cancer by GSTT1 null genotype was not statistically significant (OR = 0.72, 95%CI = 0.44–1.16, *p* = 0.185).

In controls, 6% carried both GSTM1 and GSTT1 null genotypes, and 57% were both GSTM1 and GSTT1 positive. Moreover, in lung cancer patients, 8.33% carried both GSTM1 and GSTT1 null genotype, and 56.11% had GSTM1 and GSTT1 positive. There was no statistically significant difference in the combination of both null and present genotypes of GSTM1 and GSTT1 between the two groups, which indicated no risk of lung cancer by both GSTM1 and GSTT1 null genotypes (OR = 1.41, 95%CI = 0.63–3.16, *p* = 0.421).

### 3.3. GSTM1 or GSTT1 Genotype on Risk of Lung Cancer According to Smoking Status and Family History of Cancer

The association between GSTM1 or GSTT1 genotype and lung cancer risk according to smoking status and family history of cancer is presented in Table 3. In the smoker's case, the frequency of the GSTM1 null genotype was nonsignificantly higher in lung cancer patients than in control (OR = 1.89, 95%CI = 0.96–3.71, *p* = 0.074). On the other hand, the frequency of GSTT1 null genotype was low in both smoker and nonsmoker lung cancer patients compared to control. No statistically significant increased risk of lung cancer was found in both smokers and nonsmokers based on GSTM1 and GSTT1 genotypes.

In the case of family history of cancer, although the OR for GSTM1 null genotypes was higher in study subjects, this was statistically nonsignificant (OR = 2.26, 95%Cl = 0.78–6.57, *p* = 0.155), which suggested that there was no lung cancer risk associated with the combination of GSTM1 null genotype and family history of cancer. Similarly, no statistically significant association was found between family history of cancer and null alleles of GSTT1 (OR = 0.63, 95%Cl = 0.24–1.66, *p* = 0.431).

### 3.4. Toxicity Outcomes of Platinum-Related Chemotherapy

Toxicity outcomes recorded in platinum-based chemotherapy-treated lung cancer patients are represented in Table 4. These toxicities were grouped into patients exhibiting higher toxicities (Grade 3 and Grade 4) and lower toxicities (Grade ≤ 2). The most frequent hematological toxicities included anemia, neutropenia, leukopenia, and thrombocytopenia, whereas the only nonhematological toxicity in this study was GI toxicity. However, no serious adverse events were reported throughout the study.

### 3.5. Effect of GSTM1 and GSTT1 Gene Polymorphisms on the Toxicities of Platinum-Based Chemotherapy

The role of GSTM1 and GSTT1 gene polymorphisms on platinum-based chemotherapy-induced toxicities according to toxicity grade is shown in Table 5. In the present study, no statistical significant relationship was found for GSTM1 and GSTT1 gene polymorphisms on increased risk of platinum-based chemotherapy-induced toxicities like anemia (OR = 0.70, 95%CI = 0.37–1.31, *p* = 0.336, and OR = 1.41, 95%CI = 0.68–2.92, *p* = 0.461), neutropenia (OR = 0.68, 95%CI = 0.36–1.28, *p* = 0.262, and OR = 1.53, 95%CI = 0.73–3.21, *p* = 0.274), leukopenia (OR = 0.86, 95%CI = 0.45–1.64, *p* = 0.739, and OR = 1.43, 95%CI = 0.66–3.14, *p* = 0.444), thrombocytopenia (OR = 0.91, 95%CI = 0.41–2.03, *p* = 0.838, and OR = 0.95, 95%CI = 0.38–2.41, *p* = 0.990), and gastrointestinal toxicity (OR = 0.82, 95%CI = 0.38–1.76, *p* = 0.692, and OR = 0.76, 95%CI = 0.32–1.79, *p* = 0.503) in lung cancer patients.

## 4. Discussion

As xenobiotic-metabolizing enzymes expedite eliminating a diverse array of toxic substances, their growing prominence within cancer pathophysiology underscores their pivotal role in detoxification mechanisms. It highlights their potential as targets for therapeutic interventions. Consequently, genetic polymorphisms within the enzymes intricately involved in the metabolism of carcinogens exert a significant impact on an individual's predisposition to cancer, notably lung cancer [22]. Amidst the diverse array of xenobiotic-metabolizing enzymes, the implication of GSTM1 and GSTT1 in modulating lung cancer risk stems from their potential roles in carcinogen metabolism. Despite the multitude of global studies exploring gene interactions and their association with lung cancer in various populations, the conclusions drawn from these investigations have been characterized by conflicting and nuanced findings [23].

The significance of GSTM1 and GSTT1 polymorphisms extends to their potential implications within molecular pathways, pivotal in understanding individual susceptibility to diseases such as cancer. These polymorphisms within the GST family modulate enzyme activity levels, thereby influencing the body's capacity to metabolize and eliminate toxic substances effectively. The intricate molecular pathways affected by GSTM1 and GSTT1 polymorphisms offer valuable insights into disease etiology and progression, guiding the development of personalized treatment approaches tailored to individual genetic profiles. Moreover, elucidating these pathways enhances our understanding of interindividual variability in drug metabolism and toxicity, facilitating the optimization of therapeutic regimens and advancing precision medicine initiatives.

This population-based case–control study with 180 lung cancer patients and 200 healthy controls chose to investigate the links between GST (GSTM1 and GSTT1) polymorphisms and lung cancer susceptibility in the Bangladeshi population. Beyond this, we also evaluated the effects of these genetic polymorphisms on the toxicities of platinum-based chemotherapy used in treating lung cancer patients. Our study patients and healthy controls belonged to the same ethnic background, sharing a common geographic origin. In the study subjects, the basic demographic data showed significant differences in gender, smoking status, and family history of cancer (Table 1).

Several molecular studies have elucidated crucial insights into the association between GSTM1 and GSTT1 and cancer susceptibility, revealing that individuals harboring homozygous deletions in GSTM1 and GSTT1 genes lack GST-m and GST-q enzyme activity, respectively [6]. These deletion variants offer significant utility in epidemiological cancer studies, effectively categorizing individuals into two distinct susceptibility classes based on their ability or inability to detoxify potential carcinogens through the metabolic pathways regulated by GSTM1 and GSTT1 genes. In the present study, there was a significant difference in the genotypic frequency of GSTM1 null between lung cancer patients and control and associated with a 1.6-fold increased risk of lung cancer (Table 2). This result signifies that individuals with GSTM1 null genotype have a higher risk of developing lung cancer due to decreased GST detoxification capacity, resulting in an increased concentration of carcinogens in their lung tissue. Our finding is compatible with the results reported by several studies that found a significantly increased lung cancer risk by GSTM1 null genotype in different populations [24, 25]. On the other hand, Lee et al. and López-Cima et al. reported no significant association between GSTM1 polymorphisms and lung cancer risk [26, 27]. In addition, a previous case–control study conducted in the Bangladeshi population reported that the rate of the GSTM1 null genotype was higher in the control group and found no statistically significant relationship between the individuals carrying the GSTM1 null genotype and susceptibility to lung cancer but unveiled a substantial association between the GSTP1 genotype and lung cancer [28].

In contrast to GSTM1, we found no significant relationship between GSTT1 genotype and risk of developing lung cancer (Table 2). Similarly, a meta-analysis and previous case–control studies marked no significant association between GSTT1 deletion and lung cancer [8, 29]. In contrast, an association of lung cancer risk with GSTT1 null genotype was reported in the South Indian population [30]. Moreover, in our study, no significant difference was observed in the combination of both null and present genotypes of GSTM1 and GSTT1 between the two groups (Table 2). On the contrary, Peddireddy et al. found an association between GSTM1 null/GSTT1 null combinations and lung cancer risk in the South Indian population, indicating a 2.6-fold risk of disease susceptibility [31]. However, in this study, the lack of such association in genetic polymorphisms can be ascribed to the small sample size, age differences in the study subjects (controls vs. cancer patients), and differences in clinical stages and histological classification of lung cancer.

In this population-based case–control study, we also explored the correlation of smoking status and family history of cancer with genotypes and lung cancer risk. We found no significant association between GSTM1 and GSTT1 genotype and risk of lung cancer according to smoking status (Table 3). Although the GSTM1 or GSTT1 deletion ratio was slightly higher in smokers than in nonsmokers, no statistically significant association (*p* > 0.05) was found. Similar findings by Chakova et al. revealed a statistically nonsignificant association of GSTM1 and GSTT1 deletion with lung cancer in smokers [32]. Contrasting results in several studies noticed that smokers had increased GSTM1 null deletion [33]. In the case of family history of cancer, we also observed no significant association between GSTM1 and GSTT1 genotype and risk of developing lung cancer, suggesting that family history of cancer may not be a risk factor for patients having these gene variants (Table 3).

In the second phase of this study, we found a nonsignificant trend toward an association between GSTM1 and GSTT1 gene polymorphisms and increased risk of platinum-based chemotherapy-induced toxicities like anemia, neutropenia, leukopenia, thrombocytopenia, and gastrointestinal toxicity in lung cancer patients (Table 5). Although a few studies have noted the correlation between GSTM1 and GSTT1 gene polymorphisms and platinum-based chemotherapy response in NSCLC patients [34–36], no previous studies have monitored the effects of these gene polymorphisms on chemotherapy toxicity. This result will need to be validated in prospective trials. In the case of toxicity, the lack of such association may be related to the small sample size, leading to low power to monitor the significant differences in the genotypic distribution between Grade ≤ 2 and Grade 3–4 toxicities. Besides, the use of nonplatinum drugs along with platinum agents, including paclitaxel, gemcitabine, etoposide, docetaxel, and doxorubicin, might affect the toxicity profiles. Hence, some other factors may affect platinum-induced toxicities, such as cumulative dosage, several cycles of chemotherapy, tumor molecular features, demographic characteristics, comorbidity, and intestinal bacteria.

Nevertheless, this study has several limitations and suggests future research directions for improvement. First, a formal power analysis was not conducted prior to recruitment, which may weaken the statistical robustness of the findings. Our sample size of 180 lung cancer patients and 200 healthy controls was based on practical considerations, including the availability of participants from the selected hospitals and the need to balance the study's feasibility with adequate statistical power for detecting associations. Future studies should address this by performing power analysis to determine an optimal sample size. Moreover, the absence of blinding during participant recruitment also introduces potential bias, highlighting the need to incorporate blinding procedures in future studies to enhance the accuracy of data collection and recruitment process. Additionally, our study remains focused on the genetic associations and their interaction with established lung cancer risk factors like smoking and family history, while other environmental factors like air pollution were not accounted for, which could also influence lung cancer risk. Future research should explore the interaction between environmental exposures, such as air pollution, and genetic polymorphisms (e.g., GSTM1 and GSTT1), to better understand their combined effects. Furthermore, we employed the well-established allele-specific multiplex PCR method in this study for detecting GSTM1 and GSTT1 polymorphisms, which has been validated in previous research [6, 7, 23, 27], while an additional validation method, such as PCR-RFLP, could further strengthen the genotyping results. Lastly, additional well-furnished and high-quality epidemiological studies with larger sample sizes are recommended to further validate these findings and refine the association between the studied genetic factors and lung cancer risk.

## 5. Conclusion

To conclude, the findings of this study indicate that the GSTM1 null genotype is associated with susceptibility to lung cancer, whereas the GSTT1 null genotype is not associated with an increased risk of developing lung cancer in the Bangladeshi population. Furthermore, the nonsignificant association of GSTM1 and GSTT1 gene polymorphisms with platinum-based chemotherapy-induced toxicities indicates that these gene polymorphisms are unrelated to platinum-based chemotherapy-induced toxicities. Nevertheless, our study had a limited sample size that resulted in a low power to identify minor to major genotype–disease relations and gene–environment interactions; therefore, future studies with large sample sizes are crucial to determine the actual role of genetic susceptibility in lung cancer in Bangladesh. Ultimately, by conducting additional studies in this field, it may be possible to establish a clear perception regarding genetic variations among individuals and highlight their impact on treatment outcomes and treatment-related toxicities, which will allow in the future choosing chemotherapeutic treatments according to individual genetic profiles.

## Figures and Tables

**Table 1:** Baseline demographic and clinical characteristics of the study subjects.

**Characteristics**	**Patient (** **n** = 180**)** **n (%)**	**Control (** **n** = 200**)** **n (%)**	**p** ** value**
Age (years)^a^	55.83 ± 0.66	54.70 ± 0.55	
Age distribution			
< 50 years	43 (23.88)^b^	66 (33.00)	NS^c^
> 50 years	137 (76.11)	134 (64.50)	
Gender			
Male	109 (60.56)	155 (77.50)	< 0.001
Female	71 (39.44)	45 (22.50)	
Smoking status			
Never smoker	52 (28.89)	125 (62.50)	< 0.001
Ever smoker	128 (71.11)	75 (37.50)	
Family history of cancer			
Yes	85 (47.22)	28 (14.00)	< 0.001
No	95 (52.78)	172 (86.00)	
Chemotherapy regimens			
Carboplatin + paclitaxel	57 (31.67)		
Carboplatin + gemcitabine	42 (23.33)		
Cisplatin + etoposide	34 (18.89)		
Cisplatin + paclitaxel	13 (7.22)		
Cisplatin + docetaxel	12 (6.67)		
Carboplatin + etoposide	9 (5.00)		
Carboplatin + docetaxel	7 (3.89)		
Carboplatin + doxorubicin	6 (3.33)		

*Note:p* < 0.05 was taken as the level of significance.

^a^Mean ± SEM.

^b^Numbers in parentheses show percentages.

^c^NS, not significant.

**Table 2:** GSTM1 and GSTT1 genotype frequencies in the study subjects and risk of lung cancer.

**Gene**	**Genotype**	**Patient (** **n** = 180**)** **n (%)**	**Control (** **n** = 200**)** **n (%)**	**OR (95% CI)**	**p** ** value**
GSTM1	Present	123 (68.33)	155 (77.50)	1.0 (Ref.)	
	Null	57 (31.67)	45 (22.50)	1.60 (1.01–2.52)	0.049⁣^∗^
GSTT1	Present	143 (79.44)	147 (73.50)	1.0 (Ref.)	
	Null	37 (20.56)	53 (26.50)	0.72 (0.44–1.16)	0.185
GSTM1 and GSTT1 combined	Both present	101 (56.11)	114 (57.00)	1.0 (Ref.)	
	Both null	15 (8.33)	12 (6.00)	1.41 (0.63–3.16)	0.421

Abbreviations: 95% CI, 95% confidence interval; OR, odds ratios.

⁣^∗^*p* < 0.05 considered as the level of significance.

**Table 3:** GSTM1 and GSTT1 genotype on risk of lung cancer according to smoking status and family history of cancer.

**Variable**	**Gene**	**Genotype**	**Patient (** **n** = 180**)** **n (%)**	**Control (** **n** = 200**)** **n (%)**	**OR (95% CI)**	**p** ** value**
*Smoking status*						
Smoker	**GSTM1**	Present	87 (48.33)	60 (30.00)	1.0 (Ref.)	0.074
		Null	41 (22.78)	15 (7.50)	1.89 (0.96–3.71)	
Nonsmoker		Present	36 (20.00)	95 (47.50)	1 (Ref.)	0.353
		Null	16 (8.89)	30 (15.00)	1.41 (0.69–2.89)	
Smoker	**GSTT1**	Present	103 (57.22)	57 (28.50)	1.0 (Ref.)	0.479
		Null	25 (13.89)	18 (9.50)	0.77 (0.39–1.53)	
Nonsmoker		Present	40 (22.22)	90 (45.00)	1.0 (Ref.)	0.577
		Null	12 (6.67)	35 (17.5)	0.77 (0.36–1.64)	

*Family history of cancer*						
Yes	**GSTM1**	Present	57 (31.67)	23 (11.50)	1.0 (Ref.)	
		Null	28 (15.56)	5 (2.50)	2.26 (0.78–6.57)	0.155
No		Present	66 (36.67)	132 (66.00)	1.0 (Ref.)	
		Null	29 (16.11)	40 (20.00)	1.45 (0.83–2.54)	0.242
Yes	**GSTT1**	Present	68 (37.78)	20 (10.00)	1.0 (Ref.)	
		Null	17 (9.44)	8 (4.00)	0.63 (0.24–1.66)	0.431
No		Present	75 (41.67)	127 (63.50)	1.0 (Ref.)	
		Null	20 (11.11)	45 (22.50)	0.75 (0.41–1.37)	0.375

Abbreviations: 95% CI, 95% confidence interval; OR, odds ratio.

**Table 4:** Platinum-related chemotherapy-induced toxicity according to toxicity grades.

**Toxicity**	**G** **r** **a** **d** **e** ≤ 2		**Grade 3**–**4**	
	**No.**	**%**	**No.**	**%**
Anemia	90	50.00	90	50.00
Neutropenia	97	53.89	83	46.11
Leukopenia	115	63.89	65	36.11
Thrombocytopenia	147	81.67	33	18.33
GI toxicity	141	78.33	39	21.67

*Note:* Toxicity grades are presented as two groups: Grade ≤ 2 and Grade 3–4.

**Table 5:** GSTM1 and GSTT1 genotype's effect on platinum-based chemotherapy-induced toxicities according to toxicity grades.

**Toxicities**	**Gene**	**Genotype**	**G** **r** **a** **d** **e** ≤ 2	**Grade 3**–**4**	**OR (95% CI)**	**p** ** value**
Anemia	**GSTM1**	Present	65 (72.22)	58 (64.44)	1.0 (Ref.)	—
		Null	25 (27.78)	32 (35.56)	0.70 (0.37–1.31)	0.336
	**GSTT1**	Present	69 (76.67)	74 (82.22)	1.0 (Ref.)	—
		Null	21 (23.33)	16 (17.78)	1.41 (0.68–2.92)	0.461

Neutropenia	**GSTM1**	Present	70 (72.16)	53 (63.86)	1.0 (Ref.)	—
		Null	27 (27.83)	30 (36.14)	0.68 (0.36–1.28)	0.262
	**GSTT1**	Present	74 (76.29)	69 (83.13)	1.0 (Ref.)	—
		Null	23 (23.71)	14 (16.87)	1.53 (0.73–3.21)	0.274

Leukopenia	**GSTM1**	Present	80 (69.57)	43 (66.15)	1.0 (Ref.)	—
		Null	35 (30.43)	22 (33.85)	0.86 (0.45–1.64)	0.739
	**GSTT1**	Present	89 (77.39)	54 (83.08)	1.0 (Ref.)	—
		Null	26 (22.61)	11 (16.92)	1.43 (0.66–3.14)	0.444

Thrombocytopenia	**GSTM1**	Present	101 (68.71)	22 (66.67)	1.0 (Ref.)	—
		Null	46 (31.29)	11 (33.33)	0.91 (0.41–2.03)	0.838
	**GSTT1**	Present	117 (79.59)	26 (78.79)	1.0 (Ref.)	—
		Null	30 (20.41)	7 (21.21)	0.95 (0.38–2.41)	0.990

GI toxicity	**GSTM1**	Present	99 (70.21)	24 (61.54)	1.0 (Ref.)	—
		Null	44 (31.21)	13 (33.33)	0.82 (0.38–1.76)	0.692
	**GSTT1**	Present	115 (81.56)	28 (71.79)	1.0 (Ref.)	—
		Null	28 (19.85)	9 (23.08)	0.76 (0.32–1.79)	0.503

Abbreviations: 95% CI, 95% confidence interval; OR, odds ratio.

## Data Availability

The datasets generated and/or analyzed during the current study are included in this article.
